# A Note on Elongations of Summable *QTAG*-Modules

**DOI:** 10.1155/2013/384131

**Published:** 2013-12-26

**Authors:** Alveera Mehdi, Fahad Sikander, Sabah A. R. K. Naji

**Affiliations:** ^1^Department of Mathematics, Aligarh Muslim University, Aligarh 202 002, India; ^2^Department of Mathematics, Al-Bayda University, Al-Bayda, Yemen

## Abstract

A right module *M* over an associative ring with unity is a *QTAG*-module if every finitely generated submodule of any homomorphic image of *M* is a direct sum of uniserial modules. In this paper we find a suitable condition under which a special *ω*-elongation of a summable *QTAG*-module by a (**ω**+*k*)-projective *QTAG*-module is also a summable *QTAG*-module.

## 1. Introduction and Preliminaries

The study of *QTAG*-modules was initiated by Singh [1]. Mehdi et al. studied different notions and structures on *QTAG*-modules, developed the theory of these modules by introducing several notions and some interesting properties of these modules and characterized different submodules of *QTAG*-modules. Yet there is much to explore.

Throughout this paper, all rings will be associative with unity and modules *M* are unital *QTAG*-modules. An element *x* ∈ *M* is uniform, if *xR* is a nonzero uniform (hence uniserial) module and for any *R*-module *M* with a unique composition series, *d*(*M*) denotes its composition length. For a uniform element *x* ∈ *M*, *e*(*x*) = *d*(*xR*) and *H*_*M*_(*x*) = sup⁡{*d*(*yR*/*xR*) | *y* ∈ *M*, *x* ∈ *yR*  and *y* uniform} are the exponent and height of *x* in *M*, respectively. *H*_*k*_(*M*) denotes the submodule of *M* generated by the elements of height at least *k* and *H* ^*k*^(*M*) is the submodule of *M* generated by the elements of exponents at most *k*. A *QTAG*-module *M* is called a Σ-module, if all of its high submodules are direct sums of uniserial modules. *M* is *h*-divisible if *M* = *M* ^1^ = ⋂_*k*=0_^*∞*^ *H*_*k*_(*M*).

A module *M* is summable if Soc (*M*) = ⨁_*α*<*τ*_ *S*_*α*_, where *S*_*α*_ is the set of all elements of *H*_*α*_(*M*) which are not in *H*_*α*+1_(*M*), where *τ* is the length of *M*.

A submodule *N* ⊂ *M* is nice [2, Definition 2.3] in *M*, if *H*_*σ*_(*M*/*N*) = (*H*_*σ*_(*M*) + *N*)/*N* for all ordinals *σ*; that is, every coset of *M* modulo *N* may be represented by an element of the same height.

Recall that a *QTAG*-module *M* is (*ω* + 1)-projective if there exists a submodule *N* ⊂ *H* ^1^(*M*) such that *M*/*N* is a direct sum of uniserial modules and a *QTAG*-module *M* is (*ω* + *k*)-projective if there exists a submodule *N* ⊂ *H* ^*k*^(*M*) such that *M*/*N* is a direct sum of uniserial modules [3].

A *QTAG*-module is an *ω*-elongation of a totally projective *QTAG*-module by a (*ω* + *k*)-projective *QTAG*-module if and only if *H*_*ω*_(*M*) is totally projective and *M*/*H*_*ω*_(*M*) is (*ω* + *k*)-projective.

## 2. Main Results

In [4], we studied strong *ω*-elongations of summable modules by (*ω* + *k*)-projective *QTAG*-modules. It was found that the module *M* is a special *ω*-elongation if *M* ^1^ is summable and there exists a submodule *N*⊆*H* ^*k*^(*M*) such that *M*/(*N* + *M* ^1^) is a direct sum of uniserial modules. We showed there that under certain additional circumstances on *N* these elongations are of necessity summable modules; in fact *N*∩*H*_*k*_(*M*)⊆*M* ^1^ was taken; that is, *N* is a height finite submodule of *M*.

In [5], the class of *n*-layered module was introduced which is a proper subclass of Σ-modules as follows: *M* is an *n*-layered module if *H* ^*k*^(*M*) = ⋃_*i*<*ω*_ *M*_*i*_, *M*_*i*_⊆*M*_*i*+1_⊆*H* ^*k*^(*M*), and for all *i* ≥ 1. *M*_*i*_∩*H*_*i*_(*M*)⊆*M* ^1^. It was proved there that every *n*-layered module which is a strong *ω*-elongation of a totally projective module by a (*ω* + *k*)-projective module is totally projective and vice versa; in particular each *n*-layered module is (*ω* + *k*)-projective uniquely when it is a direct sum of modules of length at most (*ω* + *n*).

Motivated by these elongations we investigate the relationship between the classes of *n*-layered modules and strong *ω*-elongations of summable modules by (*ω* + *k*)-projective modules, that is, how *n*-layered modules are situated inside these special *ω*-elongations of summable modules by (*ω* + *k*)-projective modules and whether there is an analogue with the strong *ω*-elongations of a totally projective modules by (*ω* + *k*)-projective modules.

Before doing that, we need the following preliminaries.

A module *M* is said to be pillared [6], if *M*/*M* ^1^ is a direct sum of uniserial modules. Clearly, such a module is necessarily *n*-layered module and hence a Σ-module [5], whereas the converse is not true in general. We investigate the conditions under which the converse holds good.

A module *M* is said to be a strong *ω*-elongation (of a summable module) by a (*ω* + *k*)-projective module if there exists a submodule *N*⊆*H* ^*k*^(*M*) with *M*/(*N* + *M* ^1^) a direct sum of uniserial modules. The first Ulm-factor of this module is (*ω* + *k*)-projective. There are modules with (*ω* + *k*)-projective first Ulm-factor which are not strong *ω*-elongations by a (*ω* + *k*)-projective module. These modules are with strongly (*ω* + *k*)-projective first Ulm-factor.

Now we are in the state to prove the following main result.

**Theorem 1**

An *n*-layered module is a strong *ω*-elongation by a (*ω* + *k*)-projective module if and only if it is a pillared module.

**Proof**

Suppose that *M* is an *n*-layered module, so *H* ^*k*^(*M*) = ⋃_*i*<*ω*_ *M*_*i*_, *M*_*i*_⊆*M*_*i*+1_⊆*H* ^*k*^(*M*), and for all *i* ≥ 1. *M*_*i*_∩*H*_*i*_(*M*)⊆*M* ^1^ and suppose that there exists a submodule *N* of *M* such that *N*⊆*H* ^*k*^(*M*) with *M*/(*N* + *M* ^1^) is a direct sum of uniserial modules. Clearly,

(1) $$\begin{array}{c} \frac{M/M^{1}}{\left(N+M^{1}\right)/M^{1}}≅\frac{M}{N+M^{1}}. \end{array}$$

Since *N*⊆⋃_*i*<*ω*_ *M*_*i*_, we infer that *N* = ⋃_*i*<*ω*_(*M*_*i*_∩*N*) and thus (*N* + *M* ^1^)/*M* ^1^ = ∪((*N* + *M* ^1^)/*M* ^1^) by putting *N*_*i*_ = *M*_*i*_∩*N*.

Using modular law, we compute that

(2) Ni+M1M1∩Hi(MM1)=(Ni∩Hi(M))+M1M1={0}.

Hence *M*/*M* ^1^ is a direct sum of uniserial modules and therefore *M* is pillared. The proof is complete as the converse is trivial.

**Proposition 2**

An *n*-layered module is a strong *ω*-elongation of a summable module by a (*ω* + *k*)-projective module if and only if it is a summable pillared module.

**Proof**

Suppose *n*-layered module is a strong *ω*-elongation of a summable module by a (*ω* + *k*)-projective module. Since *M* is a Σ-module and *M* ^1^ is summable, *M* has to be summable as well and Theorem 1 ensures that *M* ^1^ must be pillared.

Suppose that *M* is summable pillared module, so it ensures that *M* ^1^ is summable and pillared modules are both *n*-layered and strong *ω*-elongations by (*ω* + *k*)-projective module by taking *N* = 0, which completes the proof.

As an immediate consequence of the above, we have the following corollary.

**Corollary 3**

Suppose *M* is a Σ-module which is a strong *ω*-elongation of a summable module by a (*ω* + *k*)-projective module and the (*ω* + *n*)th Ulm-Kaplansky invariants of *M* are zero for each *n* such that 0 ≤ *n* ≤ *k* − 1, if *k* > 1. Then *M* is a summable pillared module.

**Proof**

Since (*ω* + *n*)th Ulm-Kaplansky invariants of *M* are zero, *H* ^*k*^(*M*) = *H* ^*k*^(*N*) ⊕ *H* ^*k*^(*M* ^1^) where *N* is a high submodule of *M*. Since it is a direct sum of uniserial modules, *H* ^*k*^(*N*) = ⋃_*i*<*ω*_ *N*_*i*_, *N*_*i*_⊆*N*_*i*+1_⊆*H* ^*k*^(*N*) where *N*_*i*_ ∩ *H*_*i*_(*N*) = 0. Putting *M*_*i*_ = *N*_*i*_ ⊕ *H* ^*k*^(*M*_1_), we obtain *H* ^*k*^(*M*) = ⋃_*i*<*ω*_ *M*_*i*_. Since *N* is *h*-pure in *M*, we have

(3) Mi∩Hi(M)⊆M1+Ni∩Hi(M)=M1+Ni∩Hi(M)=M1.

By Proposition 2, *M* is an *n*-layered module.

**Proposition 4**

A module of length not exceeding (*ω* + *n* − 1) is an *n*-layered module if and only if it is a direct sum of countably generated modules.

**Proof**

Suppose *M* is an *n*-layered module of length not exceeding (*ω* + *n* − 1); then *M* ^1^⊆*H* ^*n*−1^(*M*) and hence

(4) $$\begin{array}{c} \text{Soc}\left(\frac{M}{M^{1}}\right)=\underset{i<\omega }{⋂}\frac{H_{i}\left(M\right)+\text{Soc}\left(M\right)}{M^{1}}\subseteq \frac{H^{n}\left(M\right)}{M^{1}} \end{array}$$

since *H* (⋂_*i*<*ω*_(*H*_*i*_(*M*) + Soc(*M*)))⊆*M* ^1^. Moreover, we write *H* ^*n*^(*M*) = ⋃_*i*<*ω*_ *M*_*i*_, *M*_*i*_⊆*M*_*i*+1_⊆*H* ^*n*^(*M*) and *M*_*i*_∩*H*_*i*_(*M*)⊆*M* ^1^. Consequently, Soc (*M*/*M* ^1^) = ⋃_*i*<*ω*_ *T*_*i*_, where *T*_*i*_ = (*M*_*i*_ + *M* ^1^)/*M* ^1^∩Soc (*M*/*M* ^1^). We compute

(5) Ti∩Hi(MM1)=Ti∩(Hi(M)M1)=(Mi+M1)∩Hi(M)M1=(Mi∩Hi(M))+M1M1=0

hence *M* is pillared and as *M* ^1^ is bounded, *M* is a direct sum of countable modules.

The sufficiency is obvious and follows from [5].

As an immediate consequence of the above, we have the following corollary.

**Corollary**

A module *M* is an *n*-layered module if and only if each of its *H*_(*ω*+*n*−1)_(*M*)-high submodules is a direct sum of countable modules.

**Proof**

Let *M* be an *n*-layered module and *N* be its *H*_(*ω*+*n*−1)_(*M*)-high submodule. In [5], we show that *M* is an *n*-layered module precisely when *N* is an *n*-layered module. Hence using the Proposition 4, result follows immediately.

With the help of the last statement we can verify once again the validity of Corollary 3 as it is checked that a submodule *N* of *M* is *h*-high in *M* if and only if *N* is (*ω* + *n* − 1)-high in *M* whenever the (*ω* + *m*)th Ulm-Kaplansky invariants of *M* are zero for 0 ≤ *m* ≤ *n* − 1; that is,

(6) $$\begin{array}{c} \text{Soc}\left(H_{\omega }\left(M\right)\right)=\cdots =\text{Soc}\left(H_{\omega +n-1}\left(M\right)\right). \end{array}$$

A module *M* is said to be strong (*ω* + *n* − 1)-elongation of a summable module by a totally projective module if *H*_*ω*+*n*−1_(*M*) is summable and there is a nice submodule *K* of *M* such that *K*∩*H*_*ω*+*n*−1_(*M*) = 0 and *M*/(*K* ⊕ *H*_*ω*+*n*−1_(*M*)) is totally projective [3].

Now we are in the state to prove our final result.

**Theorem 6**

An *n*-layered module is a strong (*ω* + *n* − 1)-elongation of a summable module by a totally projective module if and only if it is a summable pillared module.

**Proof**

Clearly

(7) $$\begin{array}{c} \frac{M}{K⊕H_{\omega +n-1}\left(M\right)}≅\frac{M/H_{\omega +n-1}\left(M\right)}{\left(K⊕H_{\omega +n-1}\left(M\right)\right)/H_{\omega +n-1}\left(M\right)} \end{array}$$

is totally projective. Since *K*∩*H*_*ω*+*n*−1_(*M*) = 0, *K* is contained in some (*ω* + *n* − 1)-high submodule of *M*, say *N*. By Corollary 5, *N* is totally projective of length at most (*ω* + *n* − 1), so we may write *N* + ⋃_*i*<*ω*_ *N*_*i*_, where *N*_*i*_⊆*N*_*i*+1_⊆*N* and all *N*_*i*_'s are height finite in *N* and hence in *M* as *N* is isotype in *M*. Therefore

(8) K⊕Hω+n−1(M)Hω+n−1(M)  =⋃i<ω[(Ni+Hω+n−1(M)Hω+n−1(M))∩(K⊕Hω+n−1(M)Hω+n−1(M))].

In the same way, we can show that (*N*_*i*_ + *H*_*ω*+*n*−1_(*M*))/*H*_*ω*+*n*−1_(*M*) are height finite in *M*/*H*_*ω*+*n*−1_(*M*) and by [3], (*K* ⊕ *H*_*ω*+*n*−1_(*M*))/*H*_*ω*+*n*−1_(*M*) is nice in *M*/*H*_*ω*+*n*−1_(*M*) and hence *M*/*H*_*ω*+*n*−1_(*M*) is totally projective. Then

(9) $$\begin{array}{c} \frac{M}{H_{\omega }\left(M\right)}≅\frac{M/H_{\omega +n-1}\left(M\right)}{H_{\omega }\left(M\right)/H_{\omega +n-1}\left(M\right)}=\frac{M/H_{\omega +n-1}\left(M\right)}{H_{\omega }\left(M/H_{\omega +n-1}\left(M\right)\right)\;} \end{array}$$

should be a direct sum of uniserial modules and hence *M* is pillared.

On the other hand, *H*_*ω*+*n*−1_(*M*) being summable implies that *H*_*ω*_(*M*) is summable and hence *M* has to be summable; thus it is summable pillared, which completes the proof.

As an immediate consequence for *n* = 1, we have the following.

**Corollary 7**

A Σ-module is a strong *ω*-elongation of a summable module by a totally projective module if and only if it is a summable pillared module.

## References

[1] Singh S (1976). Some decomposition theorems in abelian groups and their generalizations. *Ring Theory: Proceedings of the Ohio University Conference*.

[2] Mehdi A, Abbasi MY, Mehdi F (2005). Nice decomposition series and rich modules. *South East Asian Journal of Mathematics and Mathematical Sciences*.

[3] Mehdi A, Abbasi MY, Mehdi F (2006). On (*ω*+*n*)-projective modules. *Ganita Sandesh*.

[4] Mehdi A, Sikander F, Naji Sabah ARK A study of Elongations of QTAG-Modules.

[5] Sikander F, Hasan A, Mehdi A On n-layered QTAG-modules.

[6] Mehdi F, Abbasi MY, Sirohi A (2006). On quasi isomorphic *QTAG*-modules. *Journal of Rajasthan Academy of Physical Sciences*.

